# Scalable Production of Boron Quantum Dots for Broadband Ultrafast Nonlinear Optical Performance

**DOI:** 10.3390/nano11030687

**Published:** 2021-03-09

**Authors:** Shuolei Meng, Qianyuan Chen, Hongjian Lin, Feng Zhou, Youning Gong, Chunxu Pan, Shunbin Lu

**Affiliations:** 1 International Collaborative Laboratory of 2D Materials for Optoelectronic Science & Technology of Ministry of Education, Institute of Microscale Optoelectronics (IMO), Shenzhen University, Shenzhen 518060, China; 1810285066@email.szu.edu.cn (S.M.); 1800282015@email.szu.edu.cn (H.L.); a0103368@u.nus.edu (F.Z.); 2 School of Physics and Technology, Wuhan University, 299 Bayi Rd, Wuhan 430072, China; qianyuanchen@whu.edu.cn (Q.C.); cxpan@whu.edu.cn (C.P.); 3 Shenzhen Key Laboratory of Flexible Memory Materials and Devices, Institute of Microscale Optoelectronics, Shenzhen University, Shenzhen 518000, China; nickgon@whu.edu.cn

**Keywords:** boron, saturable absorption, fast relaxation time, all-optical diode

## Abstract

A simple and effective approach based on the liquid phase exfoliation (LPE) method has been put forward for synthesizing boron quantum dots (BQDs). By adjusting the interactions between bulk boron and various solvents, the average diameter of produced BQDs is about 7 nm. The nonlinear absorption (NLA) responses of as-prepared BQDs have been systematically studied at 515 nm and 1030 nm. Experimental results prove that BQDs possess broadband saturable absorption (SA) and good third-order nonlinear optical susceptibility, which are comparable to graphene. The fast relaxation time and slow relaxation time of BQDs at 515 nm and 1030 nm are about 0.394–5.34 ps and 4.45–115 ps, respectively. The significant ultrafast nonlinear optical properties can be used in optical devices. Here, we successfully demonstrate all-optical diode application based on BQDs/ReS_2_ tandem structure. The findings are essential for understanding the nonlinear optical properties in BQDs and open a new pathway for their applications in optical devices.

## 1. Introduction

Since the discovery of graphene [1], extensive studies have been focused on graphene-analogue materials due to their remarkable physical and chemical properties [2,3]. Analogues of graphene contain carbon or other elements that possess the layered crystal structure allowing them to be exfoliated into low-dimensional formations. Among this family, boron sheets with unique trivalent electronic configuration recently have been proved to be stable in a nano-configuration and possess special properties superior to its bulk phases, potentially making them useful for electronic and optical applications [4]. 

Due to electron confinement effects, boron nanostructures may surpass graphene and carbon nanotubes for high-temperature superconductivity, high carrier mobility, massless Dirac fermions, super hardness and so on [5]. Opto-electronic properties and applications based on boron family deserve to be investigated. However, scalable production of boron quantum dots (BQDs) is limited by the covalent bonds which endow the boron with considerable strength. Technological and scientific challenge coupled with environmental considerations have prompted an exploration for a simple and efficient synthesis of BQDs. Opposite to the bottom-up growth via atomic layer deposition and molecular beam epitaxy on the Ag (111) substrate under ultrahigh vacuum condition [6,7], liquid phase exfoliation (LPE) is another effective strategy to fabricate BQDs derived from their bulk counterparts, which combines the desired advantages of super high yield, moderate price, environmental friendliness and easy processing [8,9]. On the other hand, semiconductor quantum dots (QDs) have been recognized as an advantageous nonlinear optical material over bulk and quantum well counterparts [10,11]. By virtue of the 3D confinement effect, QDs possess size-tunable emission wavelength, well-separated delta function-like density of states and large optical oscillator strength, which promises efficient nonlinear optical absorption [12]. Such quantum-mechanics-governed materials can be envisioned to develop lasers, Q-switchers and to be used as qubits in quantum computing [13,14,15]. However, the nonlinear absorption of BQDs is still unknown and worth to study.

As one of the core basic device of all-optical system and all-optical network, the all-optical diode is particularly important. Recently, an all-optical diode based on low dimensional nanomaterials has caught researchers’ attention. By assembling two nonlinear optical nanomaterials in tandem structure, it can bring non-reciprocal light transmission and form all-optical diodes, which have advantages of extremely simple structure, wide operating bandwidth, without phase matching with silicon devices and low cost [16,17,18,19]. Searching for low dimensional nanomaterials with excellent nonlinear optical absorption becomes one of the most important issues in this field. Due to size advantage and strong light interaction, these BQDs may be a candidate to form optical diodes, which can be used in integrated photonics systems [20,21,22,23]. 

Here, we report LPE synthesis of BQDs with an average diameter of 7 nm. The novel BQDs not only exhibited excellent absorption coefficient from 200 nm to 1200 nm, but also had broadband saturable absorption (SA) properties with the imaginary part of third-order nonlinear optical susceptibility $Im\chi^{\left(3\right)}\;=\;-1.29\;\times 10^{-14}\;\mathrm{esu}$, figure of merit $\mathrm{FOM}\;=\;0.710\;\times \;10^{-15}$ at 515 nm and $Im\chi^{\left(3\right)}\;=\;-2.56\;\times \;10^{-14}\;\mathrm{esu}$, $\mathrm{FOM}\;=\;2.26\;\times \;10^{-15}$ at 1030 nm. Combining BQDs with ReS_2_ (a reverse saturable absorption (RSA) material at 1030 nm), we experimentally realized passive all-optical diode, which had optical bistability and nonreciprocal light propagation. The findings validate that the proposed all-optical diode can be utilized in integrated photonics devices.

## 2. Materials and Methods

### 2.1. Preparation of the BQDs

**Figure 1:**
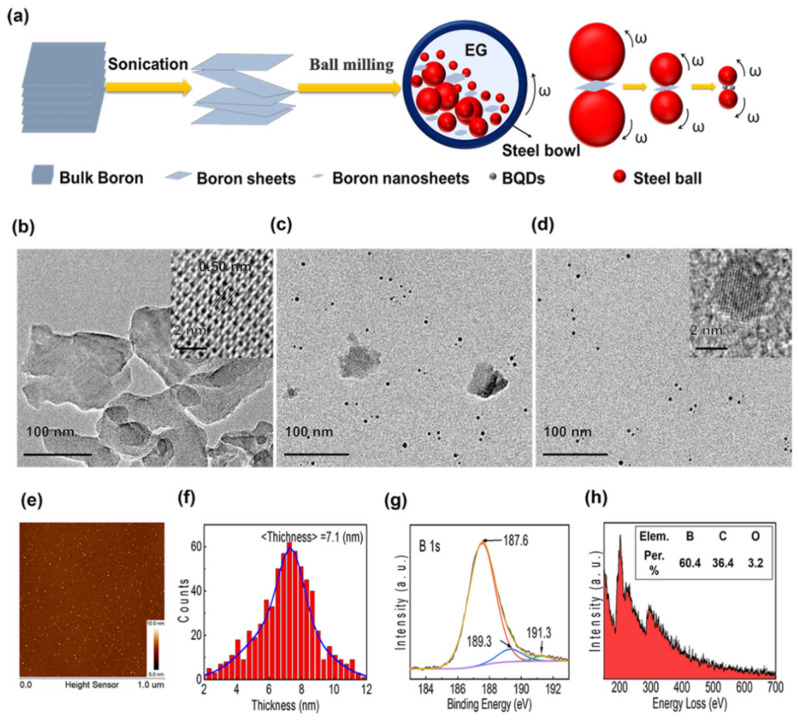
(**a**) schematic diagram of sonochemical and ball milling synthesis of boron quantum dots (BQDs). (**b**) TEM images of boron sheets obtained by sonochemical exfoliation in ethylene glycol (EG), the insets of (b) show the high resolution transmission electron microscope (HRTEM) of boron sheets. (**c**) TEM image of boron nanosheets (BNSs) and BQDs compound. (**d**) TEM images of BQDs, the inset of (d) shows the HRTEM of BQDs. (**e**) Atomic Force Microscope (AFM) characterization of the BQDs. (**f**) statistical data for BQDs showing the average thickness. (**g**) XPS spectrum (survey) for BQDs. (**h**) electron energy loss spectroscopy (EELS) studies of BQDs, the inset of (**h**) shows the corresponding atomic ratios of BQDs.

Large scale of BQDs was produced using ultra-sonication and ball milling-assisted exfoliation from bulk boron (schematically shown in Figure 1a). First, 25 mg bulk boron powder (99.99 wt %, Macklin Biochemical Co., Ltd., Shanghai, China) was directly added into 50 ml ethylene glycol (EG, ≥ 99.0 wt %, Aladdin Biochemical Technology Co., Ltd., Shanghai, China) solvent to form a suspension with an initial concentration of 0.5 mg/ml. Then, followed by bath sonication with a power of 700 W for 3 h under 5 °C in order to obtain boron sheets. The supernatant was centrifuged at 6000 rpm for 30 min to remove unexfoliated boron particles. Then, the stable light brown colored dispersions in EG were centrifuged at 15000 rpm for 60 min to concentrate boron sheets. Next, the suspension was treated with ball milling. The balls were rotated at a rate of 500 rpm for 24 h by high-energy ball milling (HEBM) (QM-3SP2, Nanjing University Instrument Plant, Nanjing, China). To obtain the BQDs and boron nanosheets compound, the as-prepared boron EG solution was firstly centrifuged at a lower (8000 rpm) speed for 30 min to obtain the supernatant, and subsequently centrifuged at a higher (13,000 rpm) speed for additional 60 min to obtain the product. The morphology and microstructure of the samples were characterized via scanning electron microscopy (SEM; Sirion, FEI Ltd., Eindhoven, Netherlands), and high resolution transmission electron microscope (HRTEM; Tecnai G2 F30) equipped with an energy-dispersive X-ray spectrometer (EDS; Genesis 7000, EDAX Inc., Philadelphia, PA, USA). The elemental compositions were analyzed via X-ray photoelectron spectroscopy (XPS; AXIS-Ultra instrument, Kratos Analytical, England) with a monochromatic Al Kα X-ray beam (225 W, 15 Ma, 15 kV). The UV-Vis diffuse reflectance spectra (DRS) of the samples were measured with the diffuse reflectance accessory of UV-Vis spectrophotometer (UV-2550; Shimadzu, Kyoto, Japan), in which BaSO4 was used as a background between 200–1200 nm.

### 2.2. Characterization of BQDs

In the planetary ball mill, the collapse of cavitation bubbles would lead to high-speed liquid micro-jets, inducing the layer gaps in bulk boron as well as the intercalation and exfoliation by the super-critical fluids. As shown in Figure 1a, balls with the diameter of 10 mm, 5 mm and 2 mm would reduce the size of boron nanosheets and ultimately grind boron nanosheets (BNSs) into BQDs. In order to measure the morphology and crystallinity of BQDs, the transmission electron microscopy (TEM) was conducted. As shown in Figure 1b, many randomly oriented few-layer boron sheets and a small number of atomically thin boron sheets were found in the EG-exfoliated bulk boron sample. The HRTEM observation (insets of Figure 1b) further demonstrated the crystalline nature of the few-layer boron sheets, which shows a clear fringe with a d-spacing of 0.50 nm, corresponding to the (104) plane of *β*-rhombohedral boron structure. After HEBM, it obtained BNSs and BQDs compound in Figure 1c [24,25]. To purify BNSs and BQDs compound, we modulated the appropriate centrifugation speed and time. As shown in Figure 1d, the size of BQDs is relatively uniform, which is consistent with the AFM results in Figure 1e. The HRTEM observation (insets of Figure 1d) further demonstrated the diameter of BQDs is around 7 nm, which is match with the statistical data of BQDs in Figure 1f. Figure 1g is binding energy spectrum characterized by XPS (VG Multilab 2000). It exhibits a high peak for binding energies ranging from 185 Ev to 195 Ev, which is believed to be the high-resolution B 1s spectra of BQDs. Furthermore, the spectra can be fitted by three Gaussian profiles that are centered at 187.6 Ev, 189.3 Ev and 191.3 Ev, respectively. This finding indicates that there are three components of bonding structures of boron. The main component at 187.6 Ev corresponds to a B-B bond, which is consistent with the reported value for bulk boron [25]. The other two components are mainly due to the oxidation of boron. The peak at 191.3 Ev can be assigned to the B-O bond in boron-rich oxide. The peak at 189.3 Ev may arise from the formation of a B-C bond due to exposure to air [25]. Results of electron energy loss spectroscopy (EELS) for BQDs are shown in Figure 1h. It exhibits a characteristic boron K-shell ionization edge at ∼188 Ev [24]. The inset of Figure 1h shows the corresponding atomic ratios of B, C and O in BQDs are 60.29 at.%, 36.44 at.% and 3.28 at.%, respectively. Small amount of O may arise from the surface contamination occurring during exposure to air atmosphere and the large amount of C may be due to the participation of carbon net substrate in the XPS measurements. 

**Figure 2:**
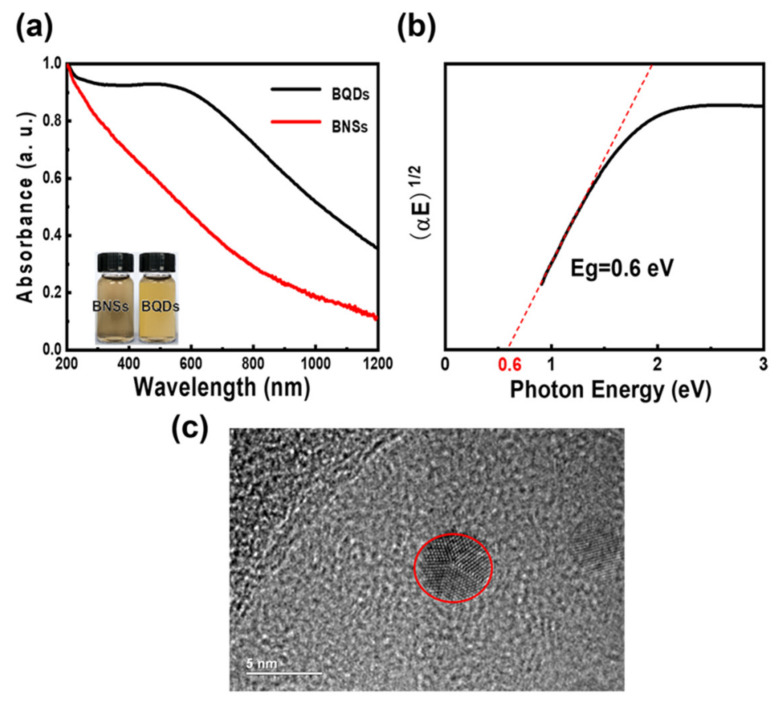
(**a**) UV-Vis-NIR absorption spectra of the BQDs and BNSs. (**b**) the corresponding optical band gap of BQDs. (**c**) the HRTEM image of BQDs with defects on surface.

The linear absorption curves are shown in Figure 2a. Compared with EG-exfoliated BNSs dispersions, BQDs dispersions exhibit stronger and broader optical absorption from 200 nm to 1200 nm. We calculated and deduced the band gap of BQDs was ~0.6 Ev with the tauc model in Figure 2b. Compared to the Hao’s work [26], our BQDs own smaller bandgap and correspondingly cannot observe photoluminescence (PL) signals at the visible band. This may be due to the high pressure and squeezing during ball milling process, defects will inevitably be introduced into the samples, which can be seen in some HRTEM image as shown in Figure 2c. On the surface of the BQDs, we can observe some folds and bulges. The effect may affect the bandgap and absorption of the BQDs. However, it owns higher and broader linear optical absorptions. The excellent optical absorption indicates strong light matter interaction in BQDs, which may lead excellent nonlinear optical property [27]. 

## 3. Results and Discussion

### 3.1. The Nonlinear Absorption of the BQDs

**Figure 3:**
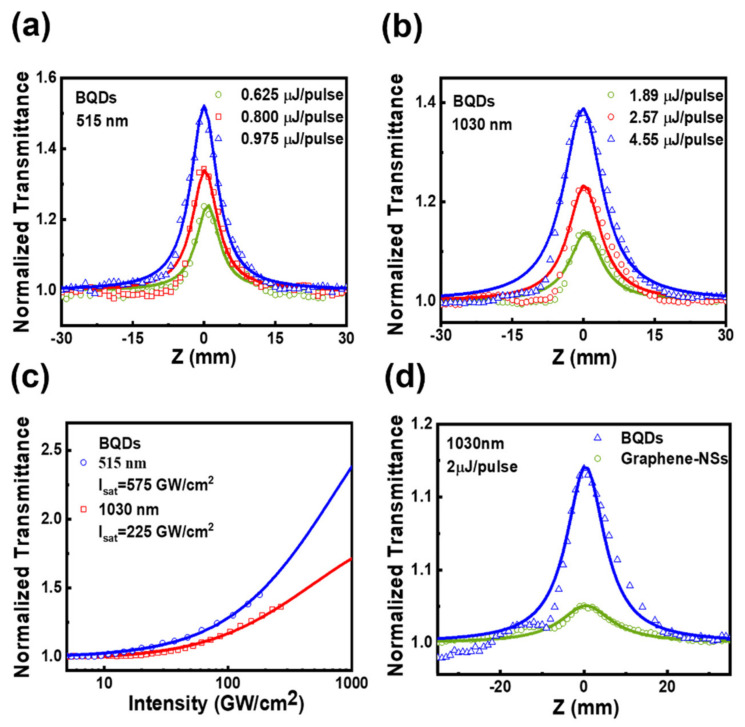
(**a**,**b**) the open aperture z-scan measurements of BQDs under different intensities at 515 nm and 1030 nm, respectively. (**c**) the normalized transmittance and input intensity of BQDs at 515 nm and 1030 nm. (**d**) the open aperture z-scan measurements of BQDs and graphene nanosheets under the excitation of 2 μJ/pulse at 1030 nm.

To investigate nonlinear optical absorption of BQDs, z-scan measurements were carried out for samples in 1-mm-quartz cuvette, the details of system are shown in Figure S1. Experimental results are shown in Figure 3, where peaks of normalized transmittance are obtained as samples move towards the focal plane (z = 0). The results in Figure 3a,b indicate that BQDs possess SA effect at both 515 nm and 1030 nm. In order to eliminate the influence from solutions (such as DMF and EG), measurements under the same condition were done to confirm that the solutions do not exhibit SA effect. As a result, the SA effect can be merely attributed to BQDs. Furthermore, thin films of BQDs were spin-coated onto 0.5-mm-quartz substrates for z-scan measurements. The results were obtained at excitation wavelengths of 515 nm and 1030 nm, confirming the SA effect of BQDs as well. With the increase of incident power, the amplitude of SA curve increases gradually, indicating that the BQDs do not produce photo-bleaching. Similarly, when the incident power decreases gradually, the amplitude also decreases and can be repeated, indicating that the BQDs did not produce photo-degradation.

To evaluate the suitability and nonlinearity of prepared BQDs as SA for ultrafast photonic devices, the most common SA model was used to fit the relationship between $T\left(z\right)$ and input peak intensity [28,29].

(1) $$T\left(z\right)=1-A_{s}⁄\left(1+I⁄I_{sat}\right)-A_{ns}$$

where $A_{s}$, I, $I_{sat}$ and $A_{ns}$ are the modulation depth, incident light intensity, saturable intensity and non-saturable loss, respectively. $\alpha_{NL}\;≅\;-\alpha_{0}/I_{sat}\;$ is fitted to be ~$10^{-11}$ cm/W at 515 nm and 1030 nm, where negative values correspond to the SA process. However, the value of $\alpha_{\mathrm{NL}}$ is affected by the concentration. To further characterize optical nonlinearities of BQDs, the imaginary part of the third-order nonlinear optical susceptibility [30] is $Im\chi^{\left(3\right)}=\left[10^{-7}c\lambda n^{2}/\left(96\pi^{2}\right)\right]\;\alpha_{NL}$, where c is the speed of light, *λ* is the excitation wavelength and n is the refractive index. Values of $Im\chi^{\left(3\right)}$ are $-1.29\;\times \;10^{-14}\;\mathrm{esu}$ at 515 nm and $-2.56\;\times \;10^{-14}\;\mathrm{esu}$ at 1030 nm. The figure of merit ($FOM=Im\chi^{\left(3\right)}/\alpha_{0}$) of BQDs for the third-order optical nonlinearity is $0.710\;\times \;10^{-15}$ at 515 nm and $2.26\;\times \;10^{-15}$ at 1030 nm which are comparable to graphene in Figure 3d, biological tellurium PmPv (Bio-Te-PmPv) [30] and black phosphorus (BP) [17] listed in Table 1, and some other materials [31,32,33,34,35,36]. The excellent nonlinear optical absorption performance of BQDs may origin from its strong light-matter interactions, which can be clearly seen from the linear absorption spectrum on Figure 2a. Compared with nanosheets, the BQDs own larger light-matter interaction area. Otherwise, the quantum confinement effect will further enhance interaction between BQDs and light [37].

### 3.2. Ultrafast Carrier Dynamics of the BQDs

For semiconductor materials, the investigation of the carrier decay dynamics is quite important to analyze the mechanism and potential for practical application in optoelectronic devices [15,20,38]. To further investigate the ultrafast carrier dynamics of BQDs, ultrafast time-resolved transient absorption spectroscopy experiments have been performed, and the details of system are shown in Figure S2.

**Figure 4:**
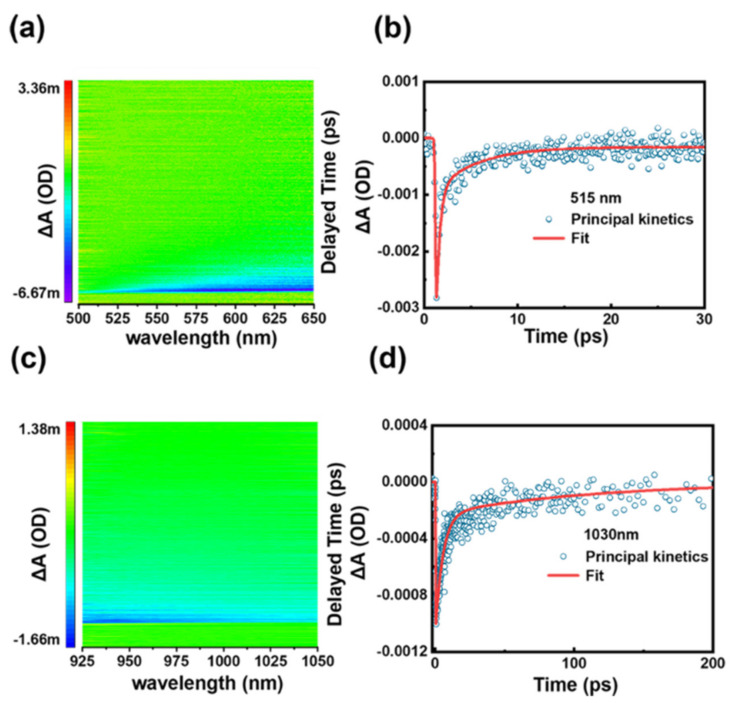
(**a**,**c**) the temporally and spectrally resolved transient absorption signal with probe light from 500 nm to 650 nm and from 925 nm to 1050 nm, respectively. (**b**,**d**) the dynamic curves of BQDs for various at 515 nm and 1030 nm, respectively.

The system was usually used to analyze the carrier relaxation process by detecting the absorption difference ΔA of the sample at different delay times. $\Delta A\;=\;A_{ex}\;-\;A_{00}$ is the absorption difference of the probe light with and without the pump light. When the BQDs were excited by the pump light, electrons would be instantly pumped to the excited state within a short period. Meanwhile the holes were left off the valence band. Then, the photo-induced carriers would be interacted with each other by the in-band carrier scattering and the thermal balance, which is referred to the fast relaxation time. After that, the photon-generated carriers would gradually cool down by interacting with photons and relax back to the ground state to recombine with the holes in a longer time, which is called the slow relaxation time. The time-resolved transient absorption spectrums of BQDs are shown in Figure 4a,c. A two-exponential model [20] $A\;=\;A_{1}exp\left(\frac{t}{\tau_{1}}\right)+A_{2}exp\left(t/\tau_{2}\right)$ could be used to fit the decay dynamics of carriers. $\tau_{1}$ and $\tau_{2}$ are the fast and slow relaxation time, respectively. $A_{1}$ and $A_{2}$ are the amplitudes of each component. The fast and slow decay time of BQDs are $\tau_{1}$ = 0.394 ps and $\tau_{2}$ = 4.45 ps at 515 nm, respectively, while those are $\tau_{1}$ = 5.34 ps and $\tau_{2}$ = 115 ps at 1030 nm (Figure 4b,d). The measured decay time of BQDs can be compared to other nanomaterials, such as BP [17] and germanium selenide (GeSe) [20]. Therefore, such a good transient response demonstrates that BQDs can be used in high-speed optics devices, such as mode locker, Q-switcher and all-optical diode.

### 3.3. All-Optical Diode Behavior

**Figure 5:**
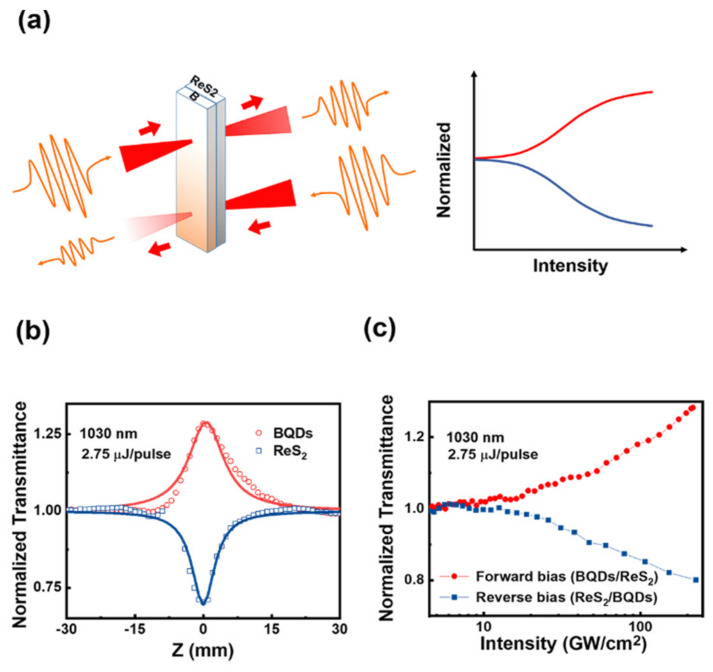
(**a**) action of an all-optical diode based on asymmetric nonlinear absorption (NLA) by employing a BQDs (saturable absorption (SA)) and ReS_2_ (reverse saturable absorption (RSA)) in tandem. (**b**) the open aperture z-scan measurement of BQDs and ReS_2_ dispersions on 2.75 μJ/pulse at 1030 nm. (**c**) the light transmission in forward bias (BQDs/ReS_2_, red circle) and reverse bias (ReS_2_/BQDs, blue square).

The spatial reciprocity of light can be represented by the linear and nonlinear light transmittance, which indicates that the transmission of light through the material in the opposite direction is same in theory. In recent studies [20,21,22], the non-reciprocal light transmission can be obtained by juxtaposing SA and RSA materials together as a tandem structure, in which transmissions of incident pulses through the structure are different in opposite directions, as shown in Figure 5a. Both SA and RSA are a result of the dynamical interaction of these media with the incident laser pulses. BQDs and ReS_2_ were fixed in z-scan system closely and BQDs was oriented towards the direction of incident light. As BQDs approached the focal point (z = 0), the light intensity increased gradually. When the light intensity is large enough, the SA effect of BQDs is first excited, the beam intensity decreases after passing through BQDs, and then passes through ReS_2_, which is not enough to stimulate a strong RSA effect. In this case, the total response will be an increase in the amount of light transmission, which is similar to the knee-voltage regime of the electron diode. Under the same experimental conditions, when the beam first passes through the ReS_2_, the RSA effect of ReS_2_ is first excited. On this occasion, the transmitted light intensity is greatly reduced, and the SA effect of BQDs cannot be excited. Therefore, the overall light transmittance decreases below the linear transmittance of the combined system while at high input intensity, which is similar the reverse bias of the electron diode. The BQDs/ReS_2_ bilayer structure has shown the same non-reciprocal transmission characteristics as the electronic diode, which is a novel all-optical diode structure. Besides, with the increase of incident light intensity, the difference between forward and reverse transmission also increases (Figure 5c). According to the extinction ratio formula, the maximum extinction ratio of this structure can be calculated as 2.5 dB. All-optical diode can limit the intensity of light and improve the transmission efficiency, which is a fundamental and extremely important link in all-optical network. In a word, the experiment simply proves the application of BQDs as saturable absorber in all-optical diode, which provides a new idea for further exploring the optical properties and practical applications of low dimensional boron materials.

As one of the core components of integrated photonics, the optical diode with a large extinction ratio, broadband operation wavelength range, and low excitation threshold have been the motivation of researchers. All optical diode of tandem structure with low dimensional boron materials provides many advantages, such as no phase-matching constraints, low cost and broadband operation wavelength range. They still face some challenges such as the small extinction ratio and a high threshold, which encourage us to investigate in the near future.

## 4. Conclusions

In conclusion, we have developed sonochemical and ball milling synthesis of BQDs dispersions. It was found that BQDs exhibited completely different optical properties from bulk boron. The experiment proved that the BQDs had a SA effect at 515 nm and 1030 nm, and had similar $Im\chi^{\left(3\right)}$ and FOM as the graphene with excellent nonlinear optical properties. Besides, the fast and slow relaxation times at different wavelengths of 515 nm and 1030 nm were components with a lifetime of about 0.394 ps ~5.34 ps and 4.45 ps ~115 ps, respectively. According to the results of transient absorption spectroscopy, BQDs not only possess ultrafast recovery times, but also exhibit SA effect. Using BQDs as saturable absorber to realize nonreciprocal all-optical diode opens a new way to explore the application of LD boron in optics. The all-optical diode with low threshold and wide operating bandwidth can be widely used in various fields such as photonic computing, all-optical networks, quantum communications and optical information processing.

## Figures and Tables

**Table 1:** Nonlinear optical coefficients of BQDs and other nanomaterials.

Laser	Sample	T (%)	$\alpha_{0}$ $\left(cm^{-1}\right)$	$I_{sat}$ $\left(GW/cm^{2}\right)$	$Im\chi^{\left(3\right)}$ $\left(\times 10^{-14}\;esu\right)$	$\mathrm{FOM}\;(\times 10^{-15})$	Ref.
515 nm, 340 fs	Bio-Te-PmPV	54.0	6.17	201±35	−(1.07±0.11)	1.74±0.18	[30]
515 nm, 340 fs	BP dispersion	86.2	1.48	N/A	−(0.49±0.05)	3.30±0.35	[17]
515 nm, 216 fs	BQDs	17.7	17.30	575±143	−(1.29±0.38)	0.71±0.19	This work
1030 nm, 340 fs	Bio-Te-PmPV	52.4	6.47	145±23	−(2.76±0.58)	4.27±0.91	[30]
1030 nm, 340 fs	BP dispersion	80.3	2.19	N/A	−(0.53±0.12)	2.40±0.49	[17]
1030 nm, 216 fs	Graphene-NSs	75.5	2.81	49±14	−(0.80±0.11)	2.84±0.41	This work
1030 nm, 216 fs	BQDs	32.3	11.30	225±64	−(2.56±0.79)	2.26±0.70	This work

## Data Availability

The data presented in this study are available on request from the corresponding author.

## Supplementary Materials

The following are available online at https://www.mdpi.com/2079-4991/11/3/687/s1, Figure S1: Schematic diagram of the Z-scan experimental setup, Figure S2: Schematic diagram of the employed non-degenerate transient absorption experiment.
