# Supplementary material for: Scalable Production of Boron Quantum Dots for Broadband Ultrafast Nonlinear Optical Performance
Source: Nanomaterials (Basel). 2021 Mar 9;11(3):687. doi: 10.3390/nano11030687 (PMC8001285; doi:10.3390/nano11030687)
Supplement: Supplementary file 1 [file nanomaterials-11-00687-s001.pdf]

nanomaterials

MDPI

Supplementary Materials

Scalable production of boron quantum dots for broadband ultrafast nonlinear optical performance

Shuolei Meng [1], Qianyuan Chen[2], Hongjian Lin [1], Feng Zhou [1], Youning Gong [3], Chunxu Pan [2] and Shunbin Lu [1,*]

[1] International Collaborative Laboratory of 2D Materials for Optoelectronic Science & Technology of Ministry of Education, Institute of Microscale Optoelectronics (IMO), Shenzhen University, 518060, Shenzhen, China; 1810285066@email.szu.edu.cn (S.M.); 1800282015@email.szu.edu.cn (H.L.); a0103368@u.nus.edu (F.Z.)
[2] School of Physics and Technology, Wuhan University, 299 Bayi Rd, 430072 Wuhan, China; qianyuanchen@whu.edu.cn (Q.C.); cxpan@whu.edu.cn (C.P.)
[3] Institute of Microscale Optoelectronics, Shenzhen Key Laboratory of Flexible Memory Materials and Devices, Shenzhen University, 518000, Shenzhen, China; nickgon@whu.edu.cn
* Correspondence: shunbin_lu@szu.edu.cn;

Z-scan technique is the common and effective method to investigate the third order nonlinear optical property of materials [1]. Here, we build z-scan system as shown in Figure S1 to study the third order NLA of BQDs dispersions and the interaction behavior of all-optical diode. The system considers the total transmittance through the sample as a function of the incident laser intensity, while the sample sequent moves through the focus of the lens (along the z-axis). For the visible and near-infrared z-scan experiments, the laser operates at 515 nm and 1030 nm with 216 fs pulse duration and 1 kHz repetition frequency. The model and the brand of the femtosecond laser are CB5-05-0085-10-0 and Light Conversion, respectively.

In the measurement process, the power of the laser is fixed, and then the sample is moved back and forth in the focus of the lens along the direction of light propagation, so as to change the intensity of the incident light before the sample by changing the size of the light spot which incident on the sample. Then, the transmittance curve of the sample under different light intensities can be obtained by recording the power change of the detector during each movement.

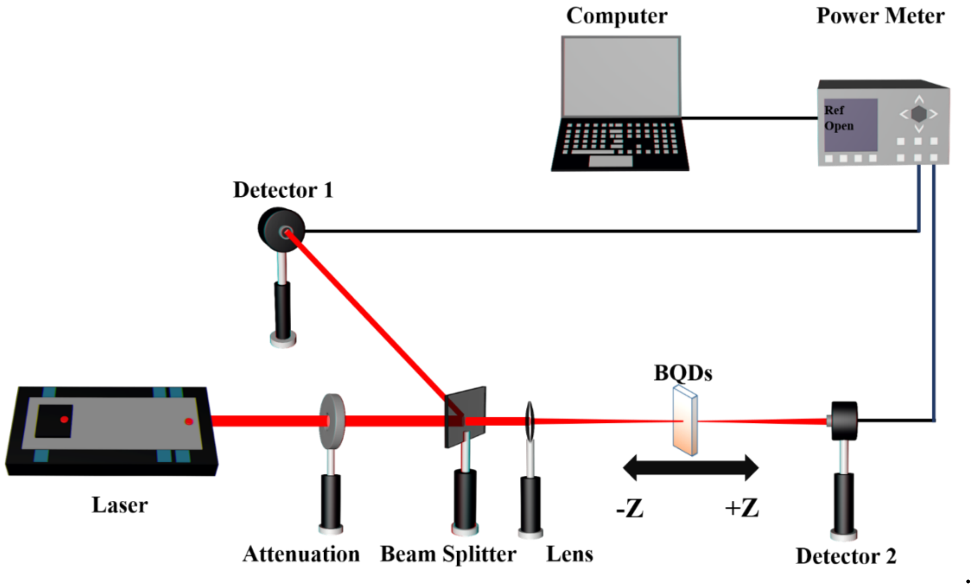

Figure S1. Schematic diagram of the Z-scan experimental setup.

The ultrafast carrier dynamics of BQDs in Figure S2 was investigated by femto-second time-resolved transient absorption (TA) spectrometer. Femtosecond pulses are generated by a mode-locked Ti: sapphire oscillator-seeded regenerative amplifier (repetition rate is 1 kHz; wavelength is 800 nm). The 800 nm pules beam is divided into two components by a 9:1 beam splitter. The major component is sent to an optical parametric amplifier (OPA, TPR-TOPAS-U, America) to generate the 400 nm pump pulse. The remaining component is sent into CaF2 crystals to generate the probe pulse (Spectrum range: 500 nm to 650 nm, 925 nm to 1050 nm). The delay time of the probe pulse and the pump pulse is automatically adjusted through a time alignment stage controlled by the computer. Subsequently, the probe pulse is detected by a spectrometer and recorded in the computer.

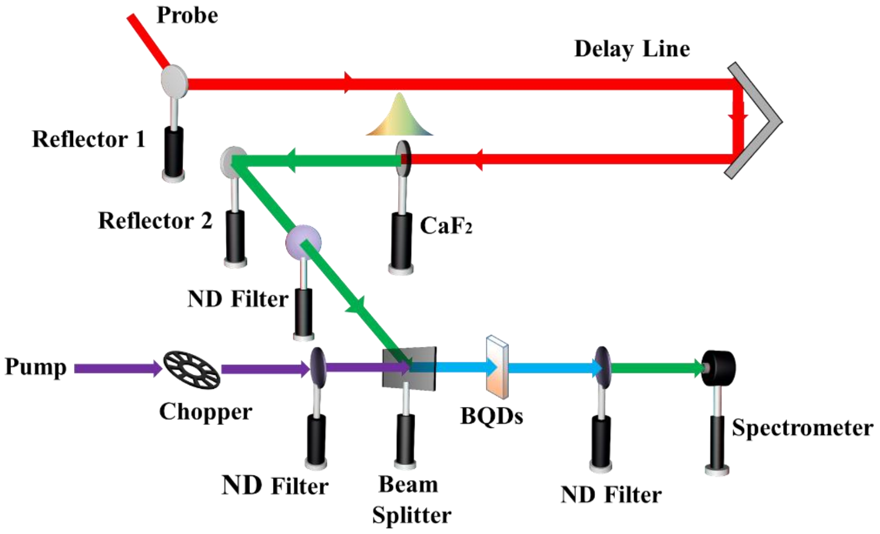

Figure S2. Schematic diagram of the employed non-degenerate transient absorption experiment.

References

1. Sheik-Bahae, M.; Said, A.A.; Wei, T.H.; Hagan, D.J.; Van Stryland, E.W. Sensitive measurement of optical nonlinearities using a single beam. *IEEE J. Quant. Electron.* ***1990***, 26(4), 760–769.
